# Clinical and biological features of gonococcal arthritis: a 20-year retrospective study

**DOI:** 10.1080/07853890.2026.2704311

**Published:** 2026-08-26

**Authors:** Zoé Gendebien, Thibaut Lejeune, Christian von Frenckell, Jean-Baptiste Giot, Cecile Meex, Sophie de Worm, Anne-Sophie Sauvage, Clio Ribbens, Olivier Malaise

**Affiliations:** ^a^ Department of Rheumatology, CHU of Liège, Belgium; ^b^ Department of Nephrology, CHU of Liège, Belgium; ^c^ Department of Infectious Diseases, CHU of Liège, Belgium; ^d^ Department of Clinical Microbiology Departments, CHU of Liège, Belgium

**Keywords:** Gonococcal arthritis, Neisseria gonorrhoeae, septic arthritis, polymerase chain reaction (PCR), disseminated gonococcal infection

## Abstract

**Objectives:**

Gonococcal arthritis is a rare but severe complication of *Neisseria gonorrhoeae* infection, often underdiagnosed due to variable presentation and difficulties of conventional tests to detect *N. gonorrhoeae*. We aimed to describe its clinical features, diagnostic yield of polymerase chain reaction (PCR) *versus* culture and patient outcomes.

**Material & Methods:**

We retrospectively analyzed all cases of gonococcal arthritis admitted to the University Hospital of Liège from January 2005 to June 2025 (*n* = 7). Patients were included if they presented with arthritis, defined either by clinical joint swelling or effusion on ultrasonography, and microbiological evidence of *N. gonorrhoeae* infection, defined as a positive PCR or culture in synovial fluid or in the blood. Clinical, microbiological and treatment data were reviewed.

**Results:**

Seven patients (57% male, mean age: 42 years) were included with confirmed gonococcal arthritis. Three joints were affected on average, most often knee, ankle, or fingers. Urogenital symptoms were reported in 43% and urogenital PCR was positive in 66%. Synovial fluid PCR was positive in all cases when performed, with shorter time to positivity than culture (1.7 vs 2.8 days), whereas blood cultures had a low rate of positivity (29%). At the end of hospitalization, no patient had achieved full recovery. At the last follow-up visit, only one patient achieved complete recovery.

**Conclusion:**

We highlight the clinical interest of systematic synovial fluid PCR: blood and synovial fluid cultures lack diagnostic yield compared to synovial fluid PCR, while synovial fluid culture also shows delayed positivity compared with the latter. We also observe that genitourinary PCR and clinical symptoms display lack positivity. Finally, despite appropriate therapy, residual joint impairment is frequent, highlighting the need for early recognition and optimized management.

## Introduction

Infection to *Neisseria gonorrhoeae* (*N.gonorrhoeae*), a gram-negative diplococcus, is a sexually transmitted infection, the second most common in Europe, after Chlamydia trachomatis and before syphilis [1]. Most women are asymptomatic, unlike men who frequently report genitourinary complaints. Extra-genital clinical manifestations are common, mainly pharyngitis, conjunctivitis or proctitis. Among articular manifestations, the disseminated form is well recognized, while suppurative arthritis occurs in less than 50% of cases [2]. Gonococcal infection may present either as septic arthritis due to direct bacterial invasion of the joint, or as disseminated gonococcal infection, also characterized by systemic symptoms such as fever, tenosynovitis, and migratory polyarthralgia. In contrast, reactive arthritis represents a sterile inflammatory response without direct bacterial invasion of the joint, making it essential to distinguish these two entities, as their pathophysiology, diagnosis, and management differ.

Our objective was to describe the clinical presentation, diagnostic yield of microbiological tools – particularly synovial fluid polymerase chain reaction (PCR) – and outcomes of patients with gonococcal arthritis managed in our tertiary center.

## Material & methods

All patients diagnosed with gonococcal arthritis and managed in the University Hospital of Liège (Belgium) from January 2005 to June 2025 were included in a retrospective analysis. Patients were included if they presented with arthritis, defined either by clinical joint swelling or by ultrasonography, defined as effusion, and microbiological evidence of *N gonorrhoeae* infection, defined as at least one of the following: (1) positive PCR in synovial fluid, (2) positive synovial fluid culture or (3) positive blood culture. Imaging and joint aspiration were not systematically required and were performed according to clinical presentation and physician decision.

The Alinity m STI Amp Kit (Abbott, USA) was used for PCR. Of note, this use of the STI Amp Kit on synovial fluid is considered as an off-label use of the test. Synovial fluid cultures were performed at least on blood and chocolate agar plates incubated for 7 days at 35 to 37 °C in 5% CO2. Synovial fluid was also inoculated in a thioglycolate enrichment broth incubated for 7 days at 35 to 37 °C air. Blood cultures were performed in BACT/ALERT FA Plus and BACT/ALERT FN Plus culture bottles (bioMérieux) or previous version of bioMerieux blood culture bottles and incubated for 5 days in a continuous-monitoring blood culture system. Antibiotic susceptibility testing was performed on positive cultures using the reference methodology (CLSI or EUCAST guidelines) in application at the time of positivity.

This study was approved by the institutional ethics committee (2025/354). Patient informed consent was waived by the institutional ethics committee due to the retrospective, non-interventional design of the study.

Clinical outcomes were defined as follows: full recovery was defined as complete resolution of pain, absence of joint swelling, and no functional limitation; favorable outcome was defined as clinical improvement without full recovery; residual impairment was defined as persistent functional limitation and/or ongoing joint symptoms at follow-up.

Continuous variables are presented as mean ± standard deviation (SD) or median (range, minimum–maximum), depending on data distribution, and categorical variables are expressed as counts and percentages. Given the small sample size, only descriptive analyses were performed.

## Results

Seven patients presented a gonococcal arthritis during the study period. There were mostly men (57%, 4/7) with a mean age (±SD) of 42 (±14) years. All patients were of Caucasian ethnicity. The average BMI was 25 (±4.3) kg/m^2^, and 29% reported tobacco use. There was no significant comorbidity nor previous rheumatologic condition. 29% (2/7) had a previous (but no concomitant) sexually transmitted infection. Risk behaviors included unprotected sex during the past year in 43% (3/7) and multiple sexual partners in 29% (2/7).

The mean number of affected joints was 2.8 (±1.7). with a median of 3 (1–5). Two patients presented with monoarthritis (1 joint), three with oligoarthritis (2–4 joints), and two with polyarthritis (≥5 joints). In the monoarthritis group, one patient had hip involvement and the other presented with ankle arthritis. In the oligoarthritis group, the first patient presented with bilateral knee arthritis associated with elbow involvement, the second had arthritis affecting two zygapophyseal joints, and the third displayed arthritis of the first metacarpophalangeal joint, the hip, and the ankle. In the polyarthritis group, one patient presented with arthritis of the first metacarpophalangeal joint, the wrist, both knees, and one sacroiliac joint, while the second patient had bilateral hip and ankle arthritis in addition to mandibular involvement (Table 1). In 71%, chills and fever were the initial symptoms. Skin involvement was observed in one patient, consisting of pustular lesions on the hand and forearm. Only 43% (3/7) declared uro-genital symptoms.

**Table 1.** Patterns of joint involvement in patients with gonococcal arthritis.

Pattern of arthritis	*n* (%)	Mean joints/patient	Median joints/patient	Details by patient (joints involved)
Monoarthritis	2 (29%)	1	1	Patient 1: hip Patient 2: ankle
Oligoarthritis	3 (43%)	3	3	Patient 3: knees (2) + elbow Patient 4: posterolateral T7/T8 and T8/T9 facet joints (2) Patient 5: 1st MCP + hip + ankle
Polyarthritis	2 (29%)	5	5	Patient 6: 1st MCP + wrist + knees (2) + sacroiliac joint Patient 7: hips (2) + ankles (2) + mandible
Overall	7 (100%)	2.8 ± 1.7 (SD)	3	

N = number of patients; T = thoracic (vertebra); MCP: metacarpophalangeal.

Diagnosis was established either by synovial fluid analysis (71%, 5/7) or by blood culture (29%, 2/7). PCR and culture testing were variably performed across patients. Synovial fluid culture without PCR was performed in two patients (2/7; 29%) and was positive in both cases (2/2). Two patients did not undergo joint aspiration but had positive blood cultures (2/7; 29%). Among the remaining patients, one had both positive synovial PCR and culture (1/7; 14%), while two had positive synovial PCR with negative culture (2/7; 29%). Blood cultures were negative in all patients with synovial fluid–based diagnosis. There was no case in which both blood and synovial samples were positive. Among patients with synovial fluid–based diagnosis, synovial fluid analysis revealed an inflammatory profile, with a mean white blood cell count of 27,000 cells/mm³ (±14,000), a mean of 89% (±2.2) neutrophils, a mean protein concentration of 56 g/L (±1.7), and a mean glucose level of 37 mg/dL (± 41). Synovial fluid PCR, performed in 43% (3/7) of cases, was positive in all instances (3/3) and showed a shorter time to positivity (mean 1.7 ± 0.6 days), than synovial culture, performed in 5 patients, positive in 3 of them (3/5; 60%) and requiring a longer time to positivity (2.8 ± 0.8 days). To note, patients with negative cultures were antibiotic-naïve at the time of sampling. Pooled PCR – combining urine samples with vaginal or rectal, and pharyngeal swabs- was positive in all cases when available (3/3), while urogenital PCR was positive in 66% (4/6) of cases when performed (Table 2). Urine cultures were performed in all patients and were negative in all cases (7/7). Genital cultures were performed in two patients and were also negative in all cases (2/2). These findings contrast with PCR results, which showed trends to higher diagnostic yield.

**Table 2.** Laboratory findings and diagnostic yield in gonococcal arthritis.

Laboratory data	
Positive synovial fluid culture (*n*)	3/7 (43%)
Positive synovial fluid culture (*n*) when performed	3/5 (60%)
Positive blood culture (*n*)	2/7 (29%)
Positive blood culture (*n*) when performed	2/7 (29%)
Positive synovial fluid PCR (*n*)	3/7 (43%)
Positive synovial fluid PCR (*n*) when performed	3/3 (100%)
Time to positivity for fluid/joint culture (days)	2.8 (±0.8)
Time to positivity for synovial fluid PCR (days)	1.7 (±0.6)
White blood cell in synovial fluid (/mm3)	26988 (±13931)
Neutrophils on synovial fluid culture (%)	89 (±2.2)
Proteins on synovial fluid culture (g/L)	56 (±1.7)
Glucose on synovial fluid culture (mg/dL)	37 (±41)
Positive urinary/genital PCR (*n*)	4/7 (57%)
Positive urinary PCR when performed (*n*)	4/6 (66%)
CRP (mg/L)	179 (±69)

Data are expressed as n patients, % or mean ± SD. PCR: polymerase chain reaction; CRP: C-reactive protein.

Antibiotics durations were highly variable, from 7 to 42 days (mean ± SD: 16 ± 13). All patients received IV ceftriaxone. Among them, two patients had received prior antibiotic therapy with a fluoroquinolone (five days before the admission, for both), and one patient subsequently received doxycycline. Antibiotic duration varied according to disease severity, site of infection, prior antibiotic exposure before hospital admission (often initiated in primary care), and infectious disease specialist recommendations, reflecting the lack of standardized guidelines for gonococcal arthritis at the time of patient management. Antibiotic susceptibility testing was available for five culture-positive cases (blood and synovial fluid) and showed a 100% preserved susceptibility to third-generation cephalosporins and azithromycin, while 100% of samples were resistant to ciprofloxacin and tetracycline. Non-steroidal anti-inflammatory drugs were administered to 71% of patients, colchicine to 14%, and systemic glucocorticoids to 29%, with a mean dose of 24 mg methylprednisolone (±11) over a mean period of 7 days (±2.8).

At the end of hospitalization, 6/7 patients showed a favorable outcome but not full recovery. Five patients had surgical management (joint lavages, performed in cases of suspected or confirmed septic arthritis with accessible joint effusion, with one patient undergoing urgent decompressive laminectomy due to an extradural collection with neurological risk). None achieved complete recovery at the end of the hospitalization. One patient died during hospitalization from acute respiratory failure due to bilateral bronchopneumonia, which was considered unrelated to gonococcal arthritis.

At first follow-up visit, with a mean duration of 39 ± 25 days, 1 patient experienced residual joint discomfort, 1 persistent joint pain, and 4 persistent functional impairment. Residual joint swelling was observed in 2 patients. Follow-up imaging, when performed, included plain radiography in one patient, which revealed coxo-femoral chondrolysis; this same patient subsequently underwent magnetic resonance imaging (MRI), showing persistent intra-articular effusion and ultimately requiring total hip arthroplasty. MRI was performed in a second patient, in whom complete resolution of the extradural medullary collection was observed. At the last known patient visit (mean: 151 ± 164 days), 2 patients reported residual discomfort, 2 had persistent functional impairment, and 1 presented with residual joint effusion. Among them, one required revision of the total hip arthroplasty. Only one patient achieved complete recovery.

To further explore factors associated with clinical outcomes, we performed a descriptive analysis of the relationship between diagnostic delay and joint damage. Given the small sample size, no formal statistical correlation analysis was performed. The two patients who developed structural joint damage had diagnostic delays of 7 and 10 days. The patient with a 7-day delay developed chondrolysis and required total hip arthroplasty 6 months later, whereas the patient with a 10-day delay underwent urgent decompressive surgery. The patient with the shortest diagnostic delay (2 days) did not develop structural joint damage; however, no radiological follow-up was available, and mild persistent symptoms (described as residual discomfort) were reported at follow-up. Among patients with intermediate diagnostic delays (7–8 days), outcomes were heterogeneous; notably, one patient with a 7-day delay did not undergo joint lavage and achieved complete recovery at 6 months, whereas others experienced persistent functional impairment without documented structural damage. The longest diagnostic delay (11 days) was not associated with structural joint damage or need for delayed surgical intervention during follow-up, although persistent functional impairment was noted. No clear relationship between treatment appropriateness and outcome could be established.

## Discussion

This article emphasizes a major complication of *N. gonorrhoeae* infection, gonococcal arthritis, which remains the leading cause of septic monoarthritis in sexually active young adults [3]. Although only 0.5% to 3% of gonococcal infections progress to disseminated infection [4], arthritis is a frequent complication [5]. Under-diagnosis is frequent due to symptoms sometimes insidious.

The disease is often polyarticular and peripheral, unlike common non-gonococcal septic arthritis. Gonococcal arthritis presents two main clinical patterns: a disseminated form (arthritis–dermatitis syndrome) and a localized suppurative form [6,7]. Suppurative arthritis usually affects a single joint but may present as oligo/polyarticular disease. In our cohort, joint involvement predominantly reflected a disseminated pattern, with a mean of three affected joints per patient, most commonly involving the knee, ankle, and hip. This arthritis is frequently accompanied by constitutional symptoms such as fever and chills (71% in our series).

Identifying the causative pathogen remains essential to distinguish true gonococcal arthritis from a post-infectious reactive arthritis. In our cohort, blood and synovial fluid cultures showed limited positivity rate, yielding positive results in only 29% and 60%, respectively, in line with previously reported data [8]. This limited performance is likely related to the difficult-to-culture nature of *N. gonorrhoeae*, which is known to grow poorly in conventional culture conditions [9]. In contrast, synovial fluid PCR demonstrated high diagnostic yield in our series, detecting the pathogen in all cases where PCR was performed. PCR also provided a shorter time to diagnostic positivity compared with conventional cultures (1.7 vs 2.8 days). These findings are in line with previously published reports, which consistently highlight the superior diagnostic performance of molecular methods for the detection of *N. gonorrhoeae* (but without an antibiogramme) [10,11]. Consistent with our findings, the positivity rate of synovial fluid culture reported in the literature ranges from 25% to 75% [6,12], whereas PCR achieves positivity rate reported between 66 and 96% [11,12], thereby enhancing both the accuracy and timeliness of diagnosis. In parallel with the limited yield of deep-site cultures, urine PCR appears to be of limited diagnostic value, with 75% of our tests returning negative results (66% positivity for genitourinary PCR). Additionally, genitourinary clinical signs could not compensate for this lack of sensitivity since only 43% of our patients exhibited them, consistent with data reported in the literature, whereas pooled PCR testing showed better diagnostic yield [12]. Systematic and ‘whole-site’ use of PCR is encouraged by the 2021 CDC guidelines [13] that state that if disseminated gonococcal infection is suspected, nucleic acid amplification test and culture specimens from all exposed urogenital and extragenital sites should be collected and processed, in addition to NAAT and culture specimens from disseminated sites of infection (e.g. synovial fluid). They also pointed out that all *N. gonorrhoeae* isolates should be tested for antimicrobial susceptibility.

In our cohort, among patients with a synovial fluid–based diagnosis, synovial fluid analysis revealed an inflammatory profile, with a mean white blood cell count of 27,000 cells/mm³ (±14,000). This is in agreement with previous studies that demonstrated that the total number of leucocytes tends to be lower in gonococcal septic arthritis than in non-gonococcal septic arthritis [14]. One recent cohort of non-gonococcal septic arthritis confirmed that the number of leucocytes in the synovial fluid is higher than in our cohort, with approximately 70,000 cells/mm³. Glucose levels also seem lower (13 mg/dL). In contrast, CRP levels seem similar (179 mg/L in our cohort, with regard to 180 mg/L [15] or 204 mg/L [16] in literature cohorts.

Clinical outcomes were suboptimal with only one complete recovery, despite joint lavage performed in most of the cases. At follow-up, persistent joint effusion was observed in a substantial proportion of patients, and one patient ultimately required total hip arthroplasty, underscoring the risk of irreversible joint damage. These findings highlight that the term ‘favorable outcome’ should be interpreted with caution, as functional impairment and reduced quality of life may persist despite adequate treatment. Although no formal statistical analysis could be performed due to the small sample size, our data do not suggest a relationship between time to diagnostic and joint outcome. Patients with similar diagnostic delays (7–10 days) showed heterogeneous clinical courses, ranging from complete recovery to severe structural damage. Moreover, follow-up duration was heterogeneous in our cohort (151 ± 164 days), reflecting the retrospective design of the study and variability in clinical follow-up. This heterogeneity in follow-up duration may limit the comparability of long-term outcomes across patients.

This case series suffers from several limitations: first, the limited number of cases restricts the conclusion that we can draw from these patients and makes difficult any statistical analysis. The number of years of analysis (20 years) also inders the comparison, as the diagnostic and therapeutic guidelines may have changed. Moreover, the monocentric aspect of this study, performed in a tertiary European hospital, limits the extrapolation of the conclusions. We also acknowledge the retrospective aspect of this study and that fact that each analysis (PCR and culture, joint and urinary analysis …) were not systematically performed. Lastly, selection criteria could also lead to a declarative bias towards microbiologically confirmed disease, as patients were selected only if culture or PCR was positive in blood or in joint aspiration.

In conclusion, while gonococcal arthritis remains a rare complication of *N. gonorrhoea* infection, the rising incidence of invasive gonococcal infections [17] highlights the need for increased awareness. Our case series highlights several points: (a) a diagnosis of direct septic arthritis (and not only reactive arthritis) should be systematically suspected when patients present with arthritis and positive uro-genital PCR; (b) synovial fluid PCR outperforms both blood and synovial fluid cultures in positivity rate and provides faster results than synovial fluid culture, highlighting the clinical interest of synovial fluid PCR; (c) genito-urinary PCR and symptoms are negative or absent in most of the case; (d) only one patient exhibited a complete full recovery, enhancing the significant morbidity of this articular condition. However, our findings should be interpreted with caution given the small sample size and retrospective design of the study.

## Data Availability

The datasets generated during and/or analyzed during the current study are available from the corresponding author on reasonable request.

## References

[1] Mitjà O, Padovese V, Folch C, et al. Epidemiology and determinants of reemerging bacterial sexually transmitted infections (STIs) and emerging STIs in Europe. Lancet Reg Health Eur. 2023;34:100742. doi:10.1016/j.lanepe.2023.100742. 37927427 PMC10625005

[2] Bardin T. Gonococcal arthritis. Best Pract Res Clin Rheumatol. 2003;17(2):201–208. doi:10.1016/s1521-6942(02)00125-0. 12787521

[3] Momodu II, Savaliya V. Septic arthritis. In StatPearls [Internet]. Treasure Island (FL): statPearls Publishing; 2023. 30844203

[4] Moran JS. Gonorrhoea. BMJ Clin Evid. 2007;2007:1604. PMC2943790 19454057

[5] O’Brien JP, Goldenberg DL, Rice PA. Disseminated gonococcal infection: a prospective analysis of 49 patients and a review of pathophysiology and immune mechanisms. Medicine (Baltimore). 1983;62:395–406. 6415361

[6] Vijayan V, Maher L. Gonococcal arthritis. In: StatPearls. Treasure Island (FL): StatPearls Publishing; 2026. 29261865

[7] García-Arias M, Balsa A, Mola EM. Septic arthritis. Best Pract Res Clin Rheumatol. 2011;25(3):407–421. doi:10.1016/j.berh.2011.02.001. 22100289

[8] Bleich AT, Sheffield JS, Wendel GD, et al. Disseminated gonococcal infection in women. Obstet Gynecol. 2012;119(3):597–602. doi:10.1097/AOG.0b013e318244eda9. 22353959

[9] Centers for Disease Control and Prevention. Recommendations for the laboratory-based detection of Chlamydia trachomatis and Neisseria gonorrhoeae—2014. MMWR Recomm Rep. 2014;63(RR-02):1–19. PMC4047970 24622331

[10] Rouanes N, Sanchez R, Cazanave C. A case of gonococcal arthritis: diagnostic difficulties and usefulness of synovial fluid PCR. Rev Med Interne. 2018;39(1):54–56. doi:10.1016/j.revmed.2017.07.004. 28844395

[11] Maldonado-Barrueco A, Sanz-González C, Rico-Nieto A, et al. Increase in gonococcal arthritis in Madrid, Spain, 2022–2023. Int J STD AIDS. 2024;35(10):831–835. doi:10.1177/09564624241254877. 38748748

[12] Moussiegt A, François C, Belmonte O, et al. Gonococcal arthritis: case series of 58 hospital cases. Clin Rheumatol. 2022;41(9):2855–2862. doi:10.1007/s10067-022-06208-w. 35590115

[13] Workowski KA, Bachmann LH, Chan PA, et al. Sexually transmitted infections treatment guidelines, 2021. MMWR Recomm Rep. 2021;70(4):1–187. doi:10.15585/mmwr.rr7004a1. PMC8344968 34292926

[14] Horowitz DL, Katzap E, Horowitz S, et al. Approach to septic arthritis. Am Fam Physician. 2011;84(6):653–660. 21916390

[15] Okuno H, Miura S, Shinagawa K, et al. Synovial-to-blood glucose ratio as a biomarker for the early diagnosis of septic arthritis of the knee in emergency medical practice: a retrospective clinical cohort study. BMC Musculoskelet Disord. 2026;27(1):25. doi:10.1186/s12891-025-09297-1. 41526897 PMC12797685

[16] Andreasen RA, Andersen NS, Just SA, et al. Prognostic factors associated with mortality in patients with septic arthritis: a descriptive cohort study. Scand J Rheumatol. 2017;46(1):27–32. doi:10.3109/03009742.2016.1164241. 27309379

[17] Sciensano. Neisseria gonorrhoeae antimicrobial resistance surveillance report of Belgium – 2024. Brussels: sciensano; 2024.

